# When do patient reported quality of life indicators become prognostic in breast cancer?

**DOI:** 10.1186/s12955-017-0834-2

**Published:** 2018-01-12

**Authors:** Chee Khoon Lee, Malcolm Hudson, John Simes, Karin Ribi, Jürg Bernhard, Alan S. Coates

**Affiliations:** 1 National Health and Medical Research Council Clinical Trials Centre, University of Sydney, Locked Bag 7, Camperdown, NSW 1450 Australia; 2 International Breast Cancer Study Group, Bern, Switzerland; 3 International Breast Cancer Study Group and Inselspital, Bern University Hospital, Bern, Switzerland

**Keywords:** Breast cancer, Quality of life, Patient reported outcomes, Linear analog self-assessment, Prognostic factors, Survival

## Abstract

**Background:**

Various patient reported quality-of-life indicators are independently prognostic for survival in metastatic breast cancer and other cancers. The same measures recorded at first diagnosis of early breast cancer carry no corresponding prognostic information. The present study aims to assess at what time in the disease evolution the prognostic association appears.

**Methods:**

Among 8024 patients enrolled in one of seven randomized controlled trials in early-stage breast cancer 3247 had a breast cancer relapse after a median follow-up of 12.1 years. Of these 677 had completed QL indicator assessments within defined windows 1, 2 or 3 months prior to relapse. We performed Cox regression analyses using these assessments and using identical instruments after relapse. All analyses were stratified by trial and adjusted for baseline clinicopathologic factors.

**Results:**

QL indicators in the months before relapse were not significantly prognostic for subsequent survival with the possibly chance exception of mood at the second month before relapse. After relapse, physical well-being was statistically significantly associated with survival (*P* < 0.001). This prognostic significance increased in later post-relapse assessments. Similar findings were observed using patient-reported indicators for nausea and vomiting, appetite, coping effort, and health perception.

**Conclusions:**

Before cancer relapse, QL indicators were not generally prognostic for subsequent survival. After relapse, QL indicators substantially predicted OS, with a stronger association later in the course of relapsed disease. Simple patient perception of disease burden seems unlikely to explain this sudden change: rather the patient’s awareness of disease relapse must contribute.

## Background

Previous studies have shown that various patient-reported quality-of-life (QL) indicators independently predict survival outcomes in metastatic breast cancer [1–5] and other cancer types [5–8]. By contrast, in studies of early-stage breast cancer, no clear relationship between QL indicators and survival has been reported [9–11], though small studies have reported associations with appetite loss [12], future perspective [13], social wellbeing [14] and with physical and functional impairment [15], while a decrease in depression was reported to be associated with longer survival [16]. The reason for this discrepancy is uncertain. If the prognostic associations in advanced disease reflect patients’ perceptions of underlying disease severity, it may well be that at initial diagnosis of early-stage disease there are no such symptoms to perceive. The timing of the emergence of prognostic association of patient-reported QL indicators is therefore important because it might illuminate the mechanism for the association of such indicators with subsequent survival. The present study uses available individual-patient data from seven International Breast Cancer Study Group (IBCSG) adjuvant therapy trials that included QL assessments. We hypothesized that there may be a lead time prior to cancer relapse during QL indicators were prognostic for subsequent survival duration. Our primary objective was to examine the association between survival and QL indicators recorded at time points shortly before each patient’s date of disease relapse, and as a secondary aim to document the time course of the prognostic significance of the same QL indicators recorded at various intervals after relapse.

## Patients and methods

Between 1986 and 2000, seven IBCSG trials randomised a total of 8024 women with operable breast cancer to different systemic treatment comparisons. Trial VI randomised premenopausal women with node-positive cancer and investigated the optimal duration and timing of adjuvant chemotherapy [17]. Trial VII [18] and trial IX [19] investigated the value of adding chemotherapy to tamoxifen to treatment for postmenopausal women with node-positive and node-negative tumors, respectively. Trial VIII investigated the role of treatment with chemotherapy, endocrine therapy comprising ovarian suppression with goserelin, and the sequential use of these modalities in premenopausal and perimenopausal women with node-negative tumors [20]. Trial 13–93 examined the introduction of a treatment gap and the value of adding tamoxifen to chemotherapy in premenopausal women [21], while Trial 14–93 investigated the role of a treatment-free gap in postmenopausal women [22]. Trial 15–95 investigated dose-intensive chemotherapy in women with high-risk, node-positive breast cancer [23].

In all of these trials, QL was measured using validated single-item linear analog self-assessment (LASA) indicators of components of QL (physical wellbeing, mood, coping, and perceived health status) shown to be affected by breast cancer, surgery, chemo- and endocrine therapy [24–27]. These were global indicators for physical well-being, mood, coping effort and perceived health status (utility). The specific indicators for side-effects were nausea/vomiting, appetite, flushing, and arm restriction. Mood and coping indicators are shown to be sensitive in identifying psychological distress, mood disorders and psychosocial dysfunction. The clinical relevance of global and specific LASA indicators has been confirmed in breast cancer trials that examined the impact of axillary clearance, chemo- and endocrine therapy, and by their association with performance status, tumor response, chemotherapy treatment benefit and toxicity, and survival duration. Each LASA indicator consisted of a 100-mm line, with scores ranged from 0 (best) to 100 (worst). The schedule of QL assessments was essentially similar in all trials, with regular early measures then additional assessment following relapse.

### Statistical methods

Only patients with documented breast cancer relapse were considered. In these patients, we examined the relationship of each QL indicator with OS using Cox regression models. All our models were stratified by trial enrolment. The hazard ratio (HR) was calculated using the estimated parameter from these models and represents the risk for a 1 point increase of the 100 point QL scale (that is, as the QL indicator worsens, the HR increases).

Our primary analysis sought any association between QL indicators and subsequent OS before cancer relapse. Survival times were measured from the landmark times before cancer relapse to the date of death (from any cause) or date of last follow-up. Three arbitrary time periods were chosen: 1, 2, and 3 months before the date of each patient’s documented relapse. QL indicators recorded within a window of 2 weeks around each of these times were analysed. In our primary analysis, all types of breast cancer relapses were considered. Multivariable analyses were performed to adjust for baseline factors at initial diagnosis (age, tumor size, estrogen receptor, performance status and axillary nodal status (node-negative vs 1–3 positive nodes vs 1–3 v 4or more positive nodes). We further performed a sensitivity analysis in which only cases with distant metastasis to skeletal, viscera, distant nodes and / or soft tissues were examined.

In our secondary analysis, we examined for the association between QL indicators at and after cancer relapse and subsequent survival. Survival times were measured from the dates of the first, second, and third QL indicator readings after relapse to the date of death (from any cause) or the date of last follow-up. The relationships between 1st, 2nd, and 3rd post-relapse QL indicators and survival from the time of relapse were tested singly using univariable Cox regression analyses stratified by trial. Multivariable analyses jointly explored first and second, and first, second, and third post-relapse QL indicators. In these multivariable analyses, survival times were measured (landmarked) from the dates of the latest QL indicator readings after relapse to the date of death. There was no adjustment for multiple comparisons. All analyses were two sided, and *P* < 0.05 was considered significant.

## Results

Of a total of 8024 patients, 3834 (47.8%) had a protocol-defined DFS event (first occurrence of breast cancer recurrence at local, regional, or distant site, contralateral breast cancer, second malignancy or death prior to a cancer event) during a median follow-up period of 12.1 years (range 0 to 21.5 years).. The patient and disease characteristics at trial entry, and QL data availability, are summarized in Table 1. Because we wanted to focus only on breast cancer relapse, we excluded 346 patients with a non-breast second primary malignancy, 223 who died without disease recurrence and 18 the nature of whose DFS event was unknown, leaving 3247 who had disease relapse in the main analytic cohort. Of these, 1243 (38%) had a relapse involving local or regional sites or contralateral breast and the remaining 2004 (62%) distant metastatic disease (Table 4 in Appendix 3).

**Table 1:** Patient and disease characteristics at trial entry, and quality of life data availability, in those with breast cancer related relapse

	Overall	Trial VI	Trial VII	Trial VIII	Trial IX	Trial 13	Trial 14	Trial 15
Patients enrolled	8024	1475	1212	1109	1669	1246	969	344
Patients who relapsed (analytic cohort)	3247	848	656	281	339	511	417	195
Characteristics in analytic cohort								
Age, median	50	44	60	44	61	43	58	46
Range	23–79	24–57	38–79	29–56	34–76	23–57	40–70	25–65
Menopausal, *n* (%)	1475 (45.4)	0 (0)	656 (100)	2 (0.7)	339 (100)	4 (0.8)	413 (99.0)	61 (31.3)
Tumor >2 cm, *n* (%)	1998 (62.8)	523 (63.2)	4 s24 (65.3)	120 (43.2)	182 (53.9)	338 (68.2)	270 (67.3)	141 (72.7)
1–3 involved axillary lymph nodes, *n* (%)	1189 (36.6)	454 (53.5)	338 (51.5)	0 (0)	1 (0.3)	250 (48.9)	146 (35.0)	0 (0)
≥ 4 involved axillary lymph nodes, *n* (%)	1438 (44.3)	394 (46.5)	318 (48.5)	0 (0)	0 (0)	261 (51.1)	270 (64.8)	195 (100)
Estrogen receptor positive, *n* (%)	2154 (66.5)	585 (69.0)	485 (73.9)	211 (75.4)	256 (76.7)	297 (58.1)	245 (58.8)	75 (38.7)
Mastectomy, *n* (%)	2258 (69.5)	637 (75.1)	543 (82.8)	130 (46.3)	182 (53.7)	327 (64.0)	291 (69.8)	148 (75.9)
Radiotherapy, *n* (%)	979 (30.2)	201 (23.7)	106 (16.2)	121 (43.1)	128 (37.8)	184 (36.0)	128 (30.7)	111 (56.9)
QL data availability at 1, 2 or 3 months before relapse^a^, *n* (%)	677 (20.9)	187 (22.1)	130 (19.8)	59 (21.0)	50 (14.7)	106 (20.7)	91 (21.8)	54 (27.7)
1 month	199	39	34	17	15	36	31	27
2 months	163	40	30	16	10	30	27	10
3 months	315	108	66	26	25	40	33	17
QL data availability at or after relapse^b^, *n* (%)	1309 (40.3)	354 (41.7)	262 (39.9)	96 (34.2)	102 (30.1)	233 (45.6)	170 (40.8)	92 (47.2)
First post-relapse reading	1309	354	262	96	102	233	170	92
Second post-relapse reading	661	188	135	57	45	125	70	41
Third post-relapse reading	202	25	25	29	22	57	31	13

*QL* quality of life

^a^at each time point before relapse, patients did not complete more than one QL assessments

^b^at each time point after relapse, patients could complete one or more QL (non-protocol) assessments

### Prognostic relevance of QL indicators measured before cancer relapse

Since the date of relapse could not be known prospectively, available QL forms in arbitrary 2-week time windows 1, 2 and 3 months prior to each patient’s data of relapse were used. Amongst 3247 patients who had disease relapse, QL forms were completed by 677 (20.9%) in one of these windows: no patient had data in more than one window (Table 1). Table 2 in Appendix 1 summarizes the distribution of QL indicators at different landmark times before and after breast cancer relapse. Table 3 in Appendix 2 shows the distribution of the different sites / types of breast cancer relapse and other DFS events.

**Fig. 1:**
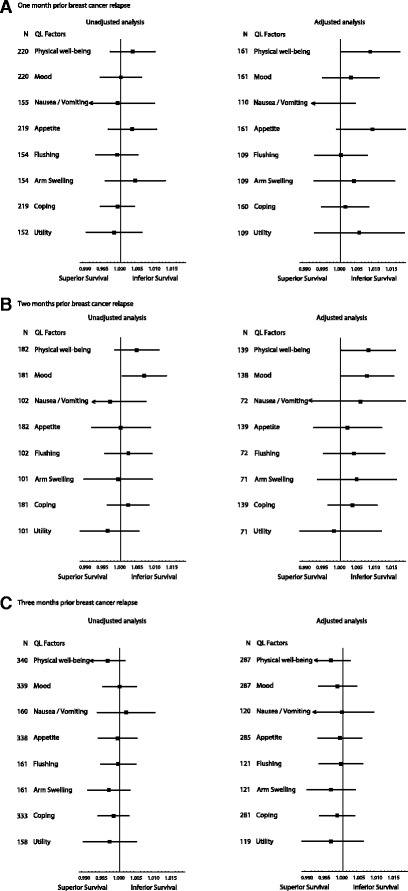
Unadjusted and adjusted hazard ratios for overall survival for 1 point scale worsening of each quality-of-life (QL) indicator at (**A**) 1 month, (**B**) 2 months, and (**C**) 3 months before breast cancer relapse. Hazard ratios for each QL indicators are represented by the squares, and the horizontal line crossing the square represents the 95% confidence interval

At 1 month and 3 months before disease relapse, none of the QL indicators showed a statistically significant relationship with subsequent OS. The results did not change significantly with adjustment for baseline factors. At 2 months before disease relapse, none of the QL indicators, except mood (HR for a 1-point change 1.008, 95% confidence interval (CI) 1.001 to 1.015, uncorrected *P* = 0.03), showed a statistically significant relationship with OS. We regard this association with mood at a single time point as a statistical artefact without biological significance. When adjusted for baseline prognostic factors, none of the QL indicators, including mood, were associated with survival (Fig. 1).

### Prognostic relevance of QL indicators measured at and beyond cancer relapse

**Fig. 2:**
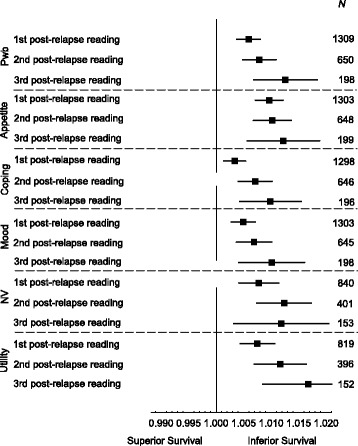
Unadjusted and adjusted hazard ratios for overall survival for 1 point scale worsening of each quality-of-life (QL) indicator after cancer relapse. PWB = physical well-being; NV = nausea and vomiting

The median survival time after disease relapse was 2.5 years (range 0 to 19.7 years). At or after disease relapse, QL forms were completed at least once by 1309 patients (40.3%) in the study cohort (Table 1). With a median time of 1.2 months after cancer relapse (first post-relapse QL indicators), physical well-being (HR per 1-point change 1.006, 95% CI 1.004 to 1.008, *P* < 0.001), was statistically significantly associated with OS. At the second post-relapse (median time 6.8 months), the corresponding HR was 1.008 (95% CI 1.005 to 1.011, *P* < 0.001) and at the third post-relapse (median time 17.8 months), 1.013 (95% CI 1.07 to 1.018, *P* < 0.001). QL indicators for nausea and vomiting, appetite, coping effort and utility taken at these time-points after cancer relapse also showed patterns similar to those for physical well-being (Fig. 2).

**Fig. 3:**
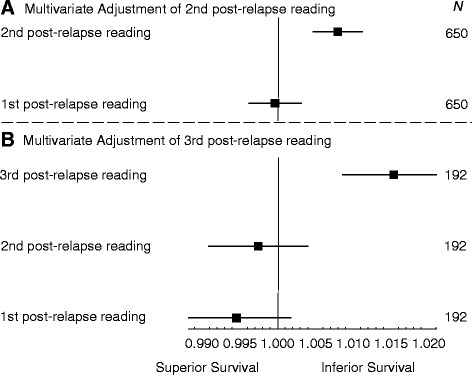
Hazard ratios for overall survival for 1 point scale worsening of physical well-being assessment when (**a**) the second post-relapse score for physical well-being was adjusted for the first post-relapse score, and (**b**) the third post-relapse score for physical well-being was adjusted for the first and second post-relapse score

When the second post-relapse QL indicators for physical well-being was adjusted for the first post-relapse QL indicators in a multivariable model, the HR for the second post-relapse QL indicators was 1.008 (95% CI 1.005 to 1.011, *P* < 0.001) while the first post-relapse QL indicators was no longer statistically significant (*P* = 0.85). The same pattern was observed among the small group who reported third post-relapse QL indicators adjusted for first and second post-relapse scores in a multivariable model (Fig. 3). These results imply that the physical well-being measured later in the evolution of recurrent disease has a stronger association with OS than earlier measurements. Similar results were observed for nausea and vomiting, appetite, coping effort, and utility (results not shown).

## Discussion

Interest in the psychological correlates of prognosis in cancer has been longstanding. Our own studies in metastatic breast cancer [28] and melanoma [29] found a consistent positive effect of “minimization” of concern. In this study, using established QL indicators which had proven prognostic in metastatic disease [2, 9] we could not demonstrate any lead-time effect, in that none of the QL indicators, (apart from the possibly chance finding of mood at 2 months), measured at 1, 2, and 3 months before cancer relapse had prognostic significance for subsequent survival. By contrast, and in keeping with prior observations, the same indicators recorded at and after cancer relapse substantially predicted subsequent survival, with stronger association for QL indicators measured later in the course of relapsed disease. The hazard ratios observed after relapse represent large effect size: every 10 points worsening of physical well-being, at the median time of 1.3 months after progression, was associated with 7% increase in hazard of death. At median times of 5.8 and 10.1 months after progression, the corresponding hazards of death were increased to 8% and 13%, respectively for every 10 points worsening of physical well-being.

This study has several strengths. It is based on individual patient QL indicator data prospectively collected in almost 4000 patients from seven adjuvant clinical trials. Among these, 22.9% had available QL indicator data falling in the period prior to the time of relapse and 40.3% had QL indicator data soon thereafter, enabling prognostic analyses to be performed at various stages of the disease trajectory around the event of disease relapse.

There are also several limitations of this study. The findings remain hypothesis-generating, as none of these clinical trials were originally designed to answer the research questions addressed in this paper. Patients were classified in these trials as having disease progression if they had local recurrence and / or more distant metastatic disease. Patient’s self-perception of the severity of the disease might differ between those who developed a local recurrence versus those with distant metastasis and hence might potentially impact on the results. However, our sensitivity analysis (Table 4 in Appendix 3) does not suggest major differences in the result of all types of relapse versus those with distant metastases only. For feasibility reasons, in these large-scale phase-III international studies where trials were conducted in different cultures with different local settings and resources, key indicators relevant to patients with breast cancer were selected as an alternative to a comprehensive QL assessment [24, 25]. Because the time of relapse could not be prospectively known, QL indicator data were only available from 22.9% of patients at the chosen time points 1, 2 or 3 months before relapse. Moreover, these data were generally from patients who relapsed early during or immediately after completion of adjuvant systemic treatments (when QL indicator assessments were scheduled) rather than those who relapsed later. We do not have detailed clinicopathologic information about the sites of relapse in these patients and have therefore not attempted to separately assess the prognostic relevance of QL indicators after relapse at various different sites. Our multivariable analyses only adjusted for baseline factors at diagnosis of early-stage breast cancer. Thus, it is possible that QL indicator data might not be prognostic for survival after relapses confined, say, to soft tissues. We used QL indicators at various time points but did not examine for effects of change in the score. However, changes in QL indicator from baseline (at the commencement of adjuvant therapy) to before and during cancer relapse may be difficult to interpret because of the significant time gap for most of these patients, and the phenomenon of response shift [30–32].

## Conclusion

In conclusion, QL indicators measured at various intervals before cancer relapse did not have prognostic significance for subsequent OS. At and after cancer relapse, QL indicators substantially predicted subsequent OS, with stronger association for QL indicators measured in the later course of relapsed disease. Patients’ self-perception of the severity of the underlying disease after relapse might be a reason for the reported QL indicators and thus contributes to their prognostic significance.

## Appendix 1

**Table 2:** Distribution of quality-of-life indicators at different landmark times before and after breast cancer relapse

	1 month prior relapse			2 months prior relapse			3 months prior relapse		
QL Indicators	N	Median^a^	IQR	N	Median^a^	IQR	N	Median^a^	IQR
Physical well-being	199	14.0	29.0	163	20.0	39.0	315	16.0	32.0
Mood	199	20.0	40.0	162	16.0	41.0	315	17.0	39.0
Nausea / Vomiting	140	3.0	7.0	90	3.0	7.0	143	3.0	13.0
Appetite	199	8.0	19.0	163	7.0	19.0	313	7.0	18.0
Flushing	139	22.0	58.0	90	27.0	70.0	144	17.5	62.0
Arm swelling	139	11.0	28.0	89	11.0	31.0	144	13.0	34.5
Coping	198	20.0	44.0	162	21.0	43.0	309	20.0	36.0
Utility	138	21.0	28.0	89	23.0	34.0	142	19.0	33.0
	1st reading post-relapse			2nd reading post-relapse			3rd reading post-relapse		
QL Indicators	N	Median	IQR	N	Median	IQR	N	Median	IQR
Physical well-being	1309	26.0	46.0	650	24.0	45.0	198	27.0	45.0
Mood	1303	27.0	46.0	645	23.0	42.0	198	22.5	41.0
Nausea / Vomiting	840	3.0	14.0	401	4.0	15.0	153	2.0	7.0
Appetite	1303	9.0	32.0	648	10.0	37.5	199	11.0	34.0
Flushing	838	19.5	49.0	398	18.0	51.0	153	23.0	54.0
Arm swelling	834	11.0	36.0	398	13.0	41.0	154	13.0	41.0
Coping	1298	35.0	50.0	646	32.0	50.0	196	31.0	53.0
Utility	819	31.0	39.0	396	33.0	35.5	152	33.0	39.0

*QL* quality of life, *IQR* interquartile range

^a^Each linear analog self-assessment QL indicators consisted of a 100-mm line, with scores ranged from 0 (best) to 100 (worst)

## Appendix 2

**Table 3:** Number of patients with different types of breast cancer relapses for each trial

Trial Number	VI	VII	VIII	IX	13	14	15	Total
	N							
No relapse	568	380	789	1118	704	495	136	4190
Local recurrence	167	102	88	84	92	72	20	625
Contralateral breast cancer	71	36	42	57	40	26	6	278
Regional nodal metastasis	106	81	16	18	59	42	18	340
Distant soft tissue / nodal metastasis	37	26	13	11	11	9	11	118
Distant bone metastasis	207	169	42	50	132	91	41	732
Distant visceral metastasis	260	242	80	119	177	177	99	1154
Second breast cancer primary	48	89	30	108	22	42	7	346
Death without recurrence	10	76	7	102	9	14	5	223
Unknown	1	11	2	2	0	1	1	18
Total	1475	1212	1109	1669	1246	969	344	8024

## Appendix 3

**Table 4:** Unadjusted analyses for (A) all types of relapses and (B) relapses limited to distant metastasis to skeletal, viscera and / or distant nodes

	(A) All types of relapses				(B) Distant relapses with skeletal, visceral and / or distant nodes			
QL indictors	N	HR	95% CI		N	HR	95% CI	
	1 month before documented relapse							
Physical well-being	199	1.004	0.997	1.010	136	1.003	0.996	1.011
Mood	199	1.001	0.994	1.007	136	0.999	0.992	1.006
Nausea / Vomiting	140	0.999	0.988	1.010	96	0.999	0.985	1.014
Appetite	199	1.002	0.995	1.009	136	1.000	0.992	1.009
Flushing	139	1.000	0.994	1.007	96	1.002	0.994	1.010
Arm swelling	139	1.005	0.997	1.014	95	1.010	1.000	1.020
Coping	198	1.000	0.994	1.005	135	1.000	0.993	1.006
Utility	138	0.998	0.989	1.007	94	0.998	0.987	1.008
	2 months before documented relapse							
Physical well-being	163	1.005	0.998	1.011	117	1.003	0.995	1.010
Mood	162	1.008	1.001	1.015	117	1.008	1.000	1.016
Nausea / Vomiting	90	0.994	0.983	1.005	66	0.993	0.981	1.005
Appetite	163	0.998	0.989	1.008	117	1.001	0.991	1.012
Flushing	90	1.003	0.995	1.010	66	1.003	0.994	1.011
Arm swelling	89	0.998	0.988	1.009	66	0.994	0.981	1.008
Coping	162	1.003	0.997	1.009	117	1.004	0.997	1.012
Utility	89	0.995	0.986	1.005	66	0.998	0.986	1.011
	3 months before documented relapse							
Physical well-being	315	0.996	0.991	1.002	189	0.994	0.988	1.001
Mood	315	0.999	0.994	1.004	189	0.997	0.991	1.003
Nausea / Vomiting	143	1.000	0.992	1.009	89	1.002	0.991	1.013
Appetite	313	0.999	0.993	1.005	187	0.998	0.990	1.006
Flushing	144	1.000	0.994	1.005	90	1.000	0.993	1.007
Arm swelling	144	0.998	0.991	1.004	90	0.998	0.991	1.005
Coping	309	0.998	0.994	1.003	184	0.996	0.990	1.002
Utility	142	0.996	0.989	1.004	88	0.995	0.986	1.005

*QL* quality-of-life, *HR* hazard ratio, *CI* confidence interval

## Abbreviations

CI: Confidence interval
DFS: Disease-free survival
HR: Hazard ratio
IBCSG: International Breast Cancer Study Group
LASA: Linear analog self-assessment
OS: Overall survival
QL: Quality-of-life
